# Two Glass Transitions Associated to Different Dynamic Disorders in the Nematic Glassy State of a Non-Symmetric Liquid Crystal Dimer Dopped with γ-Alumina Nanoparticles

**DOI:** 10.3390/ma8063334

**Published:** 2015-06-08

**Authors:** Sergio Diez-Berart, David O. López, Josep Salud, José Antonio Diego, Jordi Sellarès, Beatriz Robles-Hernández, María Rosario de la Fuente, María Blanca Ros

**Affiliations:** 1 Grup de Propietas Físiques dels Materials (GRPFM), Departament de Física i Enginyeria Nuclear, E.T.S.E.I.B. Universitat Politècnica de Catalunya, Diagonal 647, Barcelona 08028, Spain; E-Mails: david.orencio.lopez@upc.edu (D.O.L.); josep.salud@upc.edu (J.S.); 2 Dielectric Materials Physics Laboratory (DILAB), Departament de Física i Enginyeria Nuclear, E.T.S.E.I.B. Universitat Politècnica de Catalunya, Colom 11, Terrassa 08222, Spain; E-Mails: jose.antonio.diego@upc.edu (J.A.D.); jordi.sellares@upc.edu (J.S.); 3 Departamento de Física Aplicada II, Facultad de Ciencia y Tecnología, Universidad del País Vasco UPV-EHU, Apartado 644, Bilbao 48080, Spain; E-Mails: beatriz.robles@ehu.es (B.R.-H.); rosario.delafuente@ehu.es (M.R.F.); 4 Instituto de Ciencia de Materiales de Aragón (ICMA), Departamento de Química Orgánica-Facultad de Ciencias, Universidad de Zaragoza-CSIC, Zaragoza 50009, Spain; E-Mail: bros@unizar.es

**Keywords:** liquid crystal, dimer, glass transition, confinement, nanoparticles, modulated differential scanning calorimetry (MDSC), dielectric spectroscopy, thermally stimulated depolarization currents (TSDC)

## Abstract

In the present work, the nematic glassy state of the non-symmetric LC dimer α-(4-cyanobiphenyl-4′-yloxy)-ω-(1-pyrenimine-benzylidene-4′-oxy) undecane is studied by means of calorimetric and dielectric measurements. The most striking result of the work is the presence of two different glass transition temperatures: one due to the freezing of the flip-flop motions of the bulkier unit of the dimer and the other, at a lower temperature, related to the freezing of the flip-flop and precessional motions of the cyanobiphenyl unit. This result shows the fact that glass transition is the consequence of the freezing of one or more coupled dynamic disorders and not of the disordered phase itself. In order to avoid crystallization when the bulk sample is cooled down, the LC dimer has been confined via the dispersion of γ-alumina nanoparticles, in several concentrations.

## 1. Introduction

Glass transition is one of the most interesting and intriguing phenomena in the field of materials science. Any state of matter presenting some molecular dynamic disorder is susceptible to becoming a glassy state, if such molecular disorder can be frozen at a sufficiently low temperature [1,2]. For this to happen, the original state must be cooled down at a fast enough cooling rate to overcoming the phase transition to the stable, more ordered phase (thus, becoming a supercooled state) and, ultimately, get to the glass transition temperature, at which the dynamic disorder is no longer activated.

There are several experimental ways of determining the glass transition temperature. Calorimetrically, as the frozen disorder is not thermally activated, it does not contribute to the heat capacity of the sample and, therefore, there is a jump of the heat capacity from the glassy state to the supercooled state. Moreover, if the molecules are polar, the molecular dynamic modes may be detected dielectrically as relaxations. When some material is approaching the glass transition temperature, the characteristic relaxation time of the related relaxation mode that shall become frozen, gets higher and higher. As a convention, the characteristic time at the glass transition is 100 s (the related characteristic frequency is about 10 mHz). Also, regarding dielectric techniques, the so-called Thermally Stimulated Depolarization Currents (TSDC) technique is capable of identifying the glass transition temperature of glassy materials.

In the above-described situation, we shall not discard the possibility that the vitrified phase presents more than one molecular dynamic disorder. What happens in such a case? Do all the molecular disorders become frozen at the same temperature? Could it be that different disorders give rise to different glass transition temperatures? If we are dealing with structural (SG) and orientational glasses (OG), the dynamic disorder is governed by the so-called α-dielectric relaxation, which embeds the different disorders present in the vitrified phase and, therefore, there is just one glass transition temperature [3,4,5,6,7]. The main dynamic disorders represented by the α-relaxation are translational and orientational in the SG and orientational in the OG. When coming to the conformational glasses (CG), the dynamic disorders are conformational [8] or intramolecular and in the case of liquid crystalline phases, the relaxation modes can be either molecular or intramolecular [9]. Therefore, just one of the relaxations may be frozen at the glass transition [8,10] or, alternatively, all the relaxations freeze altogether [11].

Odd liquid crystal dimers are a kind of liquid crystals (LC) that are attracting considerable attention nowadays in the field of materials science, due to both their fundamental interest as well as to their potential applications in flexoelectric-based electrooptic devices. They are formed of two rigid units linked via a flexible spacer, with an odd number of carbons [12,13,14,15,16,17]. Dielectric studies show that their molecular dynamics is richer than the one of conventional LC monomers, as additional motions may appear [9,10,11,13,17,18,19,20,21,22,23]. This is the case for the non-symmetric LC dimers, such as the one studied in the present work, the so-called α-(4-cyanobiphenyl-4′-oxy)-ω-(1-pyreniminebenzylidene-4′-oxy) undecane (CBO11O.Py) [22,23,24,25]. CBO11O.Py is formed by two significantly different rigid units: A cyanobiphenyl core, which is promesogenic (*i.e.*, gives rise by itself to liquid crystalline phases) and a pyrene unit, which is bulkier than the former and was thought as a possible trigger of the glass transition of the LC dimer.

In the present study, we will show how the different dynamic disorders of CBO11O.Py do not freeze at the same temperature, and how this phenomenon is clearly detected by means of dielectric experiments. In our particular case, the identification of two glass transitions via thermal measurements is not as straightforward, even if it is also done.

One problem shall arise when performing these experiments: even if it is possible to vitrify the nematic (N) phase of the CBO11O.Py, the nematic glassy state can only be reached by cooling down the sample at high rates and, unfortunately, the supercooled N phase (N_spc_) crystallizes easily when heating up the sample from the glassy state to some temperature above, but very close to, *T*_g_ [22,23]. This implies a great technical difficulty when trying to study the dielectric properties near the glass transition. To overcome such a difficulty, the LC dimer is confined by means of the dispersion of γ-alumina nanoparticles in different concentrations. The effect of such nanoparticles in calamitic LC monomers has been recently studied by some of the authors of this paper [26]. In the confined system, CBO11O.Py + γ-alumina, crystallization is partially suppressed: the N glassy state is accessible at slower cooling rates and it remains as such when heating up to temperatures above the one where crystallization takes place in bulk CBO11O.Py. Aside from the glassy behavior of the N mesophase, the addition of these dispersive nanoparticles can also induce some other thermal and dielectric effects that must be examined. This is the case of the thermal behavior of the nematic-isotropic and crystal-nematic or crystal-isotropic phase transitions in the confined system. With respect to the dielectric properties, we will show that molecular dynamics is not considerably altered by the quenched disorder introduced by the confinement. However, the arrangement of the molecules in cells undergoes some modifications when an electric field is applied. In addition, as recently published for a LC monomer confined via the dispersion of the same type of γ-alumina nanoparticles, undesired effects due to ionic charges in the cells are diminished for the confined systems with respect to the bulk compound [26,27].

The present paper is organized as follows: In Section 2 we describe the preparation of the confined samples together with the experimental techniques. The results and discussion of the experimental data from calorimetric (MDSC) and dielectric (dielectric spectroscopy and TSDC) measurements are presented in Section 3. Finally, in Section 4, we summarize the main conclusions of the work.

## 2. Results and Discussion

**Figure 1:**
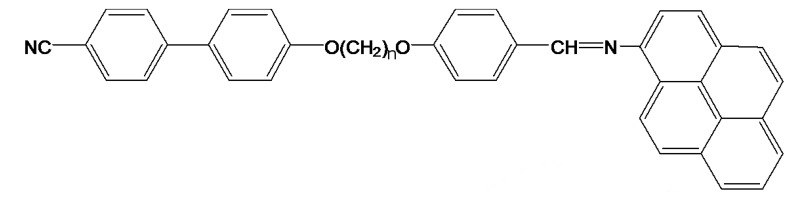
CBOnO.Py molecule.

The liquid crystal dimer, CBO11O.Py, consists of a cyanobiphenyl group attached to a pyreniminebenzylidene group via a flexible spacer of 11 methylene units, as shown in Figure 1. The interest to study the CBOnO.Py series of dimers (n accounts for the number of methylene groups), first prepared by Attard *et al.* [24], came from the combination of the mesogenic and electrooptic properties of the cyanobiphenyls and the glassy behavior of the pyrene, localized in the imine-based core. In fact, although these dimers do present such properties, for the case of our particular compound, CBO11O.Py, the nematic glassy state is only accessible under high cooling rates (15 K·min^−1^ or higher) [22]. In order to overcome this inconvenience, we introduce a quenched random disorder in the material by the dispersion of γ-alumina nanoparticles, which partially suppress the undesired crystallization of the liquid crystal dimer.

Five different samples, besides the bulk CBO11O.Py, were also considered: ρ_s_ = 0.05 g·cm^−3^, ρ_s_ = 0.16 g·cm^−3^, ρ_s_ = 0.23 g·cm^−3^, ρ_s_ = 0.31 g·cm^−3^ and ρ_s_ = 0.39 g·cm^−3^, where ρ_s_ accounts for density in grams of γ-alumina per cm^3^ of CBO11O.Py.

### 2.1. Thermal Behavior

The N phase of CBO11O.Py can be supercooled and, eventually, frozen to the corresponding nematic glassy state [22,23]. With the sample in the nematic glassy state, heating up allows us to observe the glass transition and, at about 15 K above *T*_g_, the supercooled N phase crystallizes irreversibly. Afterwards, the transitions from crystal to nematic (Cr-N) and from nematic to isotropic (N-I) take place at the corresponding transition temperatures (*T*_CrN_, *T*_NI_). These temperatures, together with *T*_g_, taken from [22,23], are listed in Table 1.

**Table 1:** Phase transition temperatures, *T*_g_, *T*_CrN_, *T*_CrI_ and *T*_NI_ for the bulk and confined system CBO11O.Py + γ-alumina. All temperatures have been recorded on heating.

ρ_s_ (g·cm−3)	*T*_NI_ (K)	*T*_CrN_ (K)	*T*_CrI_ (K)	*T*_g_ (K)
0	426.9	421.3	–	305.0
0.05	425.5	421.1	–	303.6
0.16	421.2	–	419.7	302.3
0.23	418.9	–	419.2	301.3
0.31	416.9	–	418.3	300.7
0.39	413.8	–	417.3	298.8

**Figure 2:**
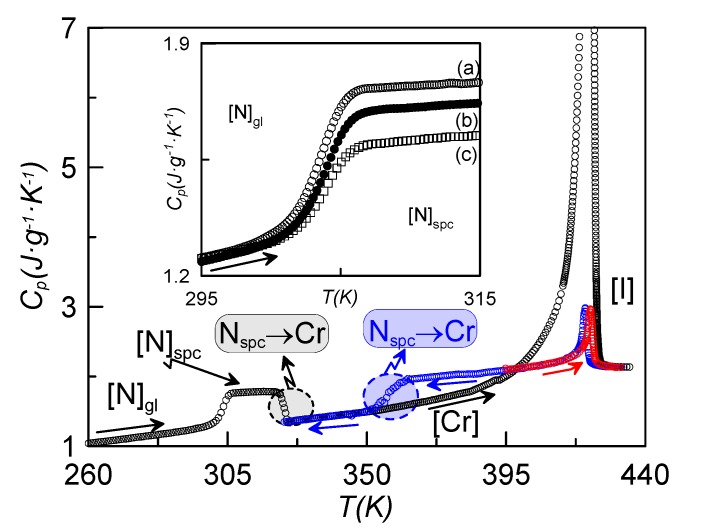
Specific heat *vs.* temperature for the ρ_s_ = 0.16 g·cm^−3^; cooling down from the isotropic phase (blue curve), heating up from the nematic phase (red curve); heating up from the N glassy state after coming from the isotropic phase at high cooling rates (black curve). The inset shows the specific heat jump at the glass transition for the sample ρ_s_ = 0.16 g·cm^−3^, after having cooled the sample down at different rates: (a) 20 K·min^−1^, (b) 15 K·min^−1^, (c) 10 K·min^−1^. All measurements were recorded at a rate of 1 K·min^−1^.

Some interesting effects arise when introducing γ-alumina nanoparticles in the host dimer. Figure 2 shows, as an example, the temperature dependence of the specific heat for the sample with ρ_s_ = 0.16 g·cm^−3^, recorded at a rate of 1 K·min^−1^, coming from different initial states. When cooling down at slow rates (1 K·min^−1^) from the isotropic phase, the I-N-Cr phase sequence is obtained (blue curve, N_spc_-Cr), with the nematic range being about 20 K larger than in the bulk sample under the same conditions of measurement. If we stop cooling at a temperature before crystallization takes place and heat the sample up from the N mesophase, the N-I phase transition takes place (red curve). If we cool the sample down from the isotropic phase at high enough rates, crystallization is avoided (or partially avoided) and we obtain the N glassy state. The black curve in Figure 2 represents the heating run from the N glassy state up to the isotropic phase. The glass transition, crystallization and melting from the crystalline state directly to the isotropic phase are clearly observed. This indicates that the N phase becomes monotropic under the influence of the γ-alumina nanoparticles. This phenomenon is observed for concentrations above 0.1 g·cm^−3^. Thus, the nematic phase is only present when cooling from the isotropic phase, and cannot be reached when heating from the crystalline state.

The inset of Figure 2 shows the characteristic specific heat jump, on heating at a rate of 1 K·min^−1^, assigned to the glass transition obtained for the sample ρ_s_ = 0.16 g·cm^−3^, previously cooled at different rates. The case denoted by (c) corresponds to the lower cooling rate (10 K·min^−1^) with a smaller specific heat jump, which gets larger as the cooling rate increases. This is understandable by the fact that, if the cooling rate is not high enough, the sample tends to crystallize partially. This behavior was already observed for other LC dimers [14]. The part remaining in the N phase ultimately becomes glassy at *T*_g_. All the more, as the concentration of nanoparticles is increased, the minimum cooling rate required to get the sample partially in the N glassy state decreases, from ~15 K·min^−1^ for bulk CBO11O.Py down to 5 K·min^−1^ for concentrations up to 0.23 g·cm^−3^ and higher (for ρ_s_ = 0.16 g·cm^−3^, this minimum cooling rate is about 10 K·min^−1^).

**Figure 3:**
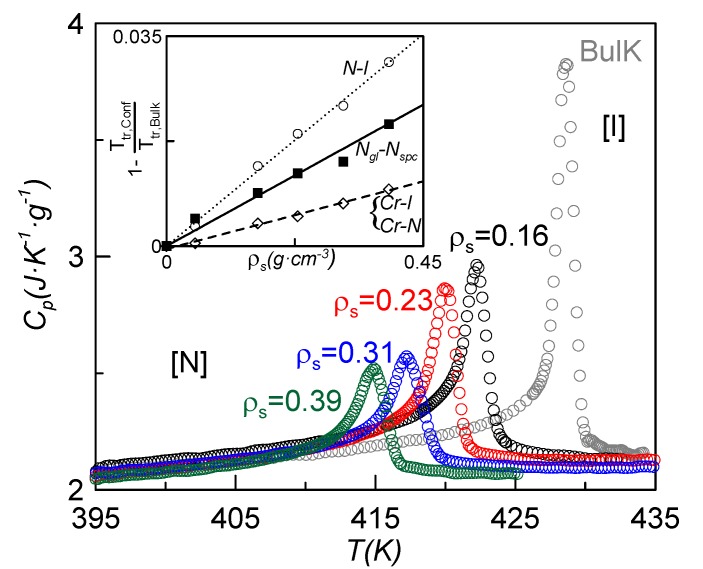
Specific heat *vs.* temperature for the six samples around the N-I phase transition. The inset shows the dependence of the absolute value of the depression of the normalized change of the phase transition temperatures (*T*_g_, *T*_CrN_, *T*_CrI_ and *T*_NI_) with concentration.

Figure 2 shows the specific heat *vs.* temperature around the N-I phase transition for bulk CBO11O.Py and some of the confined samples. As expected, the dispersion of nanoparticles induces a downward shift in the N-I phase transition temperature together with a suppressed and rounded specific heat peak similar to what is found for other liquid crystals under confinement [26,28,29,30,31]. This effect can be translated to the other phase transitions (N_g_-N_spc_, Cr-N and Cr-I), but the depression in temperature is lesser. The transition temperatures, *T*_g_, *T*_CrN_, *T*_CrI_ and *T*_NI_, are listed in Table 1 for the different samples. The dependence with concentration of the absolute value of the depression of the phase transition temperatures divided by the bulk transition temperature (from now on, normalized change of the phase transition temperature) is represented in the inset of Figure 3. These values show a linear dependence with the nanoparticles concentration of the normalized change of the N-I phase transition temperature. Such a linear dependence is fitted to the relationship

(1) $$1-\frac{T_{{\text{ρ}}_{s}}}{T_{\text{bulk}}}=A{\text{ρ}}_{\text{s}}$$

where *A* = 0.078 cm^3^·g^−1^. This behavior differs substantially from that of the 4O.8 monomer confined via the dispersion of the same γ-alumina nanoparticles [26], in which the dependence was not linear. Such a difference could be explained by arguing that, since the CBO11O.Py molecule is larger and more flexible than the 4O.8 one, more dispersive material is needed to induce similar changes in the transition temperatures in the former with respect to the latter. Taking into account concentrations of γ-alumina below 0.1 g·cm^−3^ in 4O.8, the normalized change of temperature with concentration is well fitted by a linear law [Equation (1)], with a value of *A* equal to 0.28 cm^3^·g^−1^, about 3.6 times higher than the one corresponding to CBO11O.Py. This confirms the exposed argument. When the concentration of γ-alumina in 4O.8 goes over 0.1 g·cm^−3^, the influence of the marginal nanoparticle is less every time and a kind of saturation takes place, giving rise to a non-linear relationship [26]. In the case of CBO11O.Py, the used concentrations of γ-alumina are not enough to saturate.

The inset of Figure 3 also shows the normalized change for the melting and glass transition temperatures *vs.* nanoparticle concentration, which can also be fitted by a linear relationship as Equation (1). The values of the fitting constants *A* are 0.023 cm^3^·g^−1^ for the melting temperature and 0.051 cm^3^·g^−1^ for the glass transition temperature. These values are about 1/3 and 2/3, respectively, of the corresponding value for the N-I phase transition. The isotropic and nematic phases are much more fluid than the N glassy state. These differences in viscosity make the N-I phase transition more sensitive to the influence of the presence of γ-alumina than the glass transition. The crystal to fluid-like phase transition seems to be affected by confinement in a lesser extent than the N_gl_-N_spc_ phase transition. In our opinion, the order of the phase plays an important role.

### 2.2. Dielectric Measurements

The dielectric behavior of bulk CBO11O.Py far from the glass transition has already been published [22,23]. The crystallization of the sample 75 K above *T*_g_ prevents a dielectric study close to the glass transition. The partial suppression of crystallization by the dispersion of γ-alumina nanoparticles in the host LC allows us to study the LC dimer dielectrically, reducing the forbidden temperature gap. Nevertheless, confinement could alter the molecular dynamics of the bulk liquid crystal and, therefore, a comparison is carried out below. Among the prepared confined samples, the one with ρ_s_ = 0.23 g·cm^−3^ is chosen for our dielectric study, because higher concentrations have a similar impact in the suppression of crystallization.

**Figure 4:**
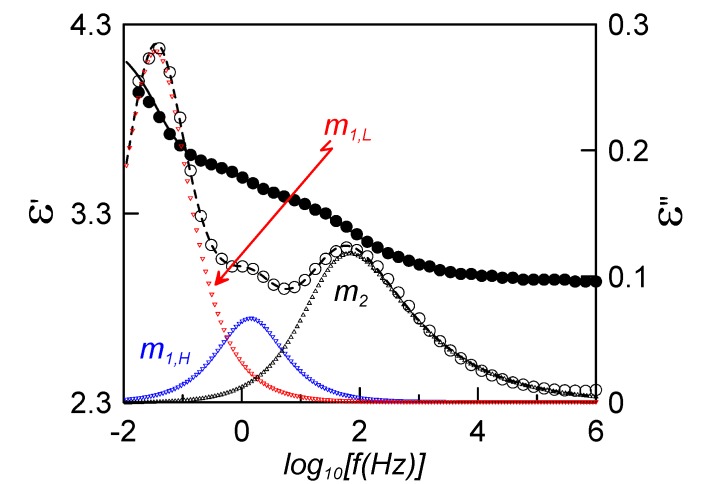
Frequency dependence of the complex dielectric permittivity for the sample ρ_s_ = 0.23 g·cm^−3^ at 313 K (N mesophase) under a DC bias of 20 V. Full circles account for the experimental real part, empty circles for the experimental imaginary part; fittings to Equation (2) are shown by the continuous (real part) and dashed (imaginary part) lines. Triangles represent the deconvoluted relaxation modes from the fitting.

Figure 4 shows the real (full circles) and imaginary (empty circles) parts of the complex dielectric permittivity in the nematic phase at 313 K, for the confined sample with a DC bias of 20 V. It must be said that if no bias is applied, the sample adopts a mixed alignment, whereas the application of the DC bias increases the molecular alignment parallel to the probing electric field (homeotropic-like alignment).

Solid lines in Figure 4 correspond to the fittings of experimental data to the empirical function:

(2) $$\text{ε}\left(\text{ω}\right)=\underset{k}{\sum }\frac{\Delta {\text{ε}}_{\text{k}}}{{\left[1+{\left(i{\text{ωτ}}_{\text{k}}\right)}^{{\text{α}}_{\text{k}}}\right]}^{{\text{β}}_{\text{k}}}}+{\text{ε}}_{\infty }-i\frac{{\text{σ}}_{\text{DC}}}{{\text{ωε}}_{0}}$$

where *k* accounts for the relaxation modes present in the phase and each one is fitted according to the Havriliak-Negami function; Δε_k_ and τ_k_ are the dielectric strength and the relaxation time related to the frequency of maximum dielectric loss, respectively; α_k_ and β_k_ are parameters that describe the shape (width and symmetry) of the relaxation spectra; ${\text{ε}}_{\infty }$ is the dielectric permittivity at high frequencies (but lower than those corresponding to atomic and electronic resonance phenomena); and σ_DC_ is the electric conductivity. With respect to the α_k_ and β_k_ parameters, both are equal to 1 in the simplest (Debye) model. If the relaxation is more complex, the shape of the peaks may get broader (α_k_ < 1) and/or asymmetric (β_k_ ≠ 1). The system in the homeotropic-like alignment presents three relaxation modes in the N phase, just as the bulk material [22]. Thus, the explanation of these modes will be in the same way, according to Sttochero’s theoretical model for the dielectric behavior of non-symmetric LC dimers [20]. According to this model, the lowest frequency mode, m_1L_, is identified due to the flip-flop motion of the pyrene group, which immediately forces the cyanobiphenyl unit to reorient and, therefore, it can be detected dielectrically. The intermediate frequency mode, m_1H_, is related to the flip-flop reorientation of the cyanobiphenyl unit. The highest frequency mode, m_2_, is due to precessions of the cyanobiphenyl group around the nematic director. Far from the glass transition, the two modes with lower frequencies, m_1L_ and m_1H_, are Debye-like. The high frequency mode, m_2_, is Cole-Cole (α_2_ < 1 and β_2_ = 1), with the parameter α_2_ ranging from ~0.8 to ~0.5 as the compound is cooled down from the isotropic phase.

The shape of the relaxation modes changes deeply when approaching the glass transition. The molecular reorientations experiment huge steric couplings and become more cooperative. Both *α*_1L_ and *α*_1H_ decrease to 0.9, while the change is more obvious in the high frequency mode, where there is not just a modification in width but also in symmetry (α_2_ ~ 0.8 and β_2_ ~ 0.5). This phenomenon is typical for glass-forming materials when approaching the glass transition [4,8].

**Figure 5:**
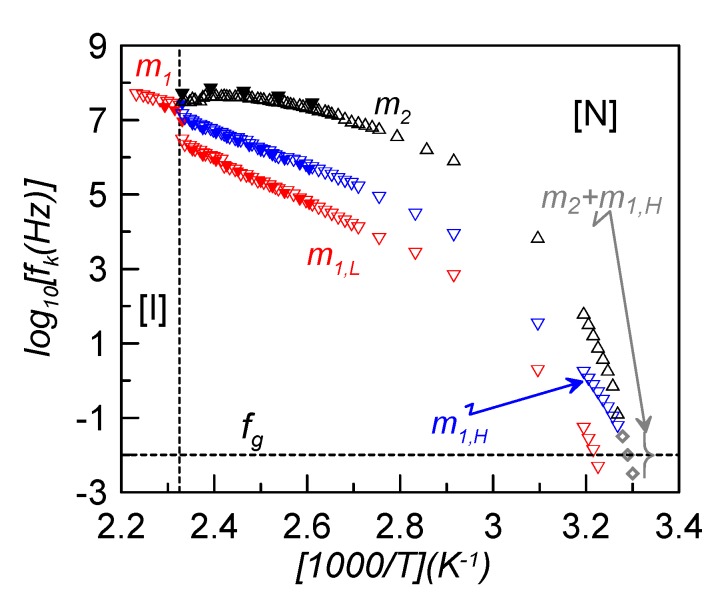
Arrhenius plot of the relaxation frequencies of the different elementary contributions for the sample ρ_s_ = 0.23 g·cm^−3^ (empty symbols). For comparison purposes, results for bulk CBO11O.Py from reference [22] are also shown (full symbols).

In the present study, the main interest consists in evaluating how the relaxation frequency of the maximum dielectric loss changes with temperature. This is presented in the Arrhenius plot of Figure 5, where some values for bulk CBO11O.Py (full symbols) are also shown for comparison [22]. First of all, it can be seen how the frequencies of bulk and confined samples do not differ significantly, so the dispersion of γ-alumina nanoparticles does not seem to noticeably affect the relaxation frequency of the modes.

For dielectric relaxations, a glass transition (denoted as dynamic glass transition) is obtained when the characteristic relaxation time, *τ_k_*, reaches about 100 s, or the corresponding frequency is approximately 10 mHz. As is clearly observed in Figure 5, m_2_ and m_1H_ relaxation modes tend to overlap in frequency when approaching the glass transition. At a low enough temperature, both modes are indistinguishable and the observed dielectric peak is attributed to a superposition of both. The fitting of this complex mode (grey symbols in Figure 5) leads to a very asymmetric Cole-Davidson relaxation (α = 1, β = 0.3). This behavior is comparable to that found for the symmetric LC dimer, CB7CB, in which the twist-bend nematic (N_TB_) mesophase is frozen and, at the glass transition, the two observed relaxation modes overlap [11]. However, in the present case, the low frequency mode, m_1L_, is observed separated from the other two and gets frozen at a temperature about 7 K higher. This would mean that, from a dielectric point of view, two glass transition temperatures involving different molecular motions seem to be present. As the lower in temperature glass transition is due to the dynamic disorder of the cyanobiphenyl group and the higher one is due to the pyrene reorientations, we denote both glass transition temperatures as *T*_gCB_ ~ 304 K and *T*_gP*y*_ ~ 311 K. The nematic environment at these low temperatures makes the steric interactions of each group much stronger in the case of the pyrene reorientations, which become more hindered and need more thermal energy to be activated and, accordingly, *T*_gPy_ > *T*_gCB_.

The question that now arises is if it is possible to certify the existence of this double glass transition by other experimental techniques.

### 2.3. Thermal Stimulated Depolarization Currents (TSDC)

The unexpected dielectric results near the glass transition leads us to make a complementary analysis by means of Thermal Stimulated Depolarization Currents (TSDC). This technique is particularly well-suited for this analysis because it is equivalent to a very low frequency dielectric measurement [32]. TSDC measurements were performed by means of the so-called non-isothermal windowing (NIW) polarization method, in which the poling field is applied during a cooling ramp [33,34]. In our measurements the samples were cooled from the isotropic phase down to 325 K (*T*_i_) at a fast cooling rate (30 K·min^−1^). They were held at this temperature for one minute and then cooled down to the deposit temperature (*T*_d_ = 275 K) at 2.5 K·min^−1^. Within this second ramp, a static poling field was applied to polarize the material, from an initial temperature T_p1_ to a final temperature *T*_p2_ < *T*_p1_. The sample was held at the deposit temperature for 15 minutes and, afterwards, it was heated again up to 325 K (*T*_f_) at 2.5 K·min^−1^. During the heating ramp, the depolarization current was registered.

**Figure 6:**
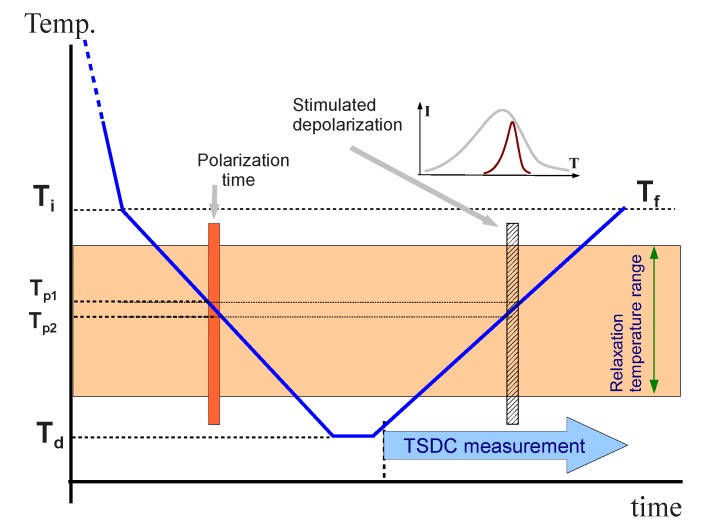
Diagram of a TSDC measurement.

This experimental technique allows the deconvolution of a complex relaxation spectrum into its elementary components, using a methodology known as the Relaxation Map Analysis (RMA) [33,34]. In a RMA, elementary spectra can be obtained if the electric field is applied only in a very small temperature range during the cooling ramp. By this way only those motions (modes) that are activated at the poling temperature can lead to some polarization at the end of the cooling ramp. So, the obtained depolarization current obtained may be associated with the elementary mode activated at the poling temperature. The entire relaxation spectrum results from the superposition of the elementary modes obtained at different polarization temperatures. In a polymer, for example, it is possible to analyze the dominant structural relaxation, the α relaxation, which accounts for the global molecular reorientations, and resolve it into its simpler components [33,34]. Figure 6 shows the thermal diagram of a TSDC measurement.

**Figure 7:**
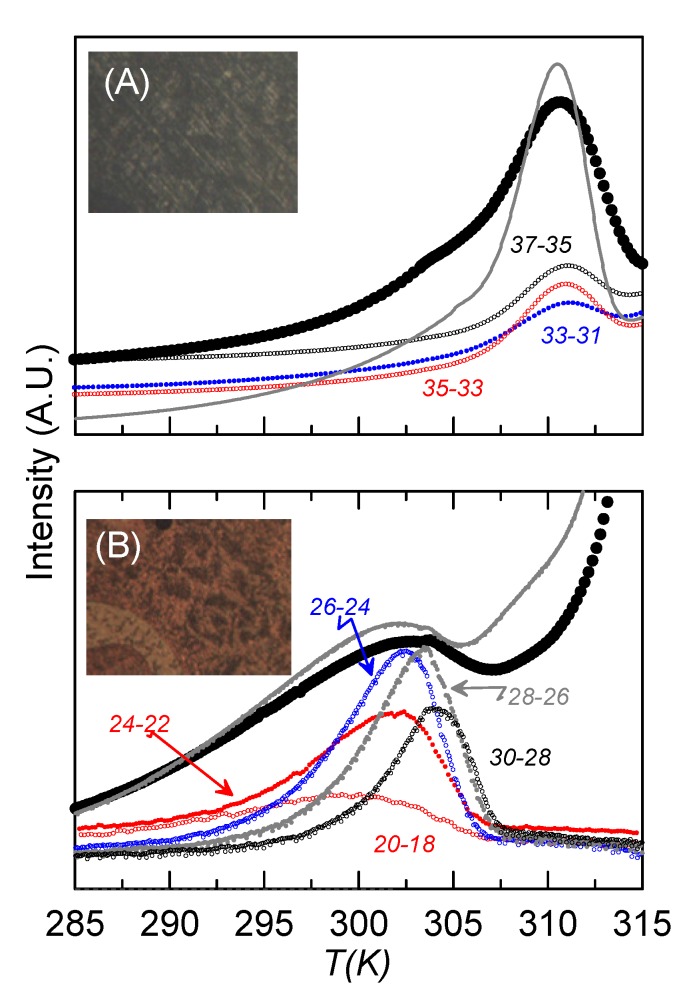
TSDC curves *vs*. temperature for the sample ρ_s_ = 0.23 g·cm^−3^ for (**A**) homeotropic-like and (**B**) mixed alignments. Big black circles represent the “complete” curves. Small circles account for some of the NIW curves for each alignment. The name of each NIW curve is “*T*_p1_-*T*_p2_”. Textures for each alignment from the polarizing microscope are also shown. Grey lines account for the “complete” curves of the bulk under similar conditions.

In this work, we apply the TSDC technique to our confined LC dimer, in order to confirm the dielectric results. In the RMA analysis, we perform different NIW measurements, changing *T*_p1_ and *T*_p2_ in each experiment, but maintaining *T*_i_ = *T*_f_ = 325 K and *T*_d_ = 275 K. Two sets of experiments have been performed, one with a poling field of 1.6 MV·m^−1^, in which the sample is in a mixed alignment, and the other one with a poling field of 12 MV·m^−1^, where the sample is more homeotropic-like, as can be observed from textures in Figure 7. In the first set of measurements, we apply the polarization field in a wide range of temperatures (*T*_p1_ = *T*_i_, *T*_p2_ = 290 K), what we call the “complete” measurement. Later, we perform the RMA analysis by several NIW measurements in which *T*_p1_−*T*_p2_ = 2 K, from the highest *T*_p1_ (=*T*_i_) until the lowest one (*T*_p1_ = 292 K), covering the “complete” range. In the second set of measurements, in order to assure the homeotropic-like molecular alignment, we have to apply the poling field from the isotropic phase. So, this field is applied from isotropic (448 K) down to 290 K in the complete measurement. In the RMA analysis the poling field is applied from isotropic (448 K) down to 325 K and then removed, before applying it again in the corresponding NIW.

Together with the textures obtained from the microscope, Figure 7 shows the complete (large circles) and some of the NIW-RMA experimental curves (small circles) of the depolarization current *vs*. temperature for the sample with ρ_s_ = 0.23 g·cm^−3^, for both homeotropic-like (Figure 7A) and mixed (Figure 7B) alignments. The complete curves obtained by TSDC can be associated to dielectric loss measurements at an extremely low frequency [32], even below that corresponding to the glass transition (10 mHz). Confirming the results from dielectric spectroscopy, two main relaxations can be observed at well-defined temperatures, which differ in about 7 K, as happened in the dielectric measurements at the glass transition. The one at a lower temperature (302 K) should be the overlap of m_2_ and m_1H_, as it corresponds to higher frequencies at the same temperature. This mode is higher in strength when the sample is less homeotropic-like, which is coherent with the fact that m_2_ should be residual in homeotropic alignments and the strength of m_1H_ at such low temperatures is very small. The other one (at 309 K), with a much higher strength when the sample is homeotropic-like, should be m_1L_. The complete curves for the bulk sample are also present in Figure 7 (grey lines), to show how the results are qualitatively similar for bulk and confined samples.

A further confirmation of the good agreement between dielectric and TSDC results comes from the fitting of the TSDC relaxation modes (RMA analysis) to some phenomenological models from which the activation energies can be obtained. If a relaxation mechanism has first order kinetics, the calculated depolarization current can be obtained from the equation

(3) $$J\left(T\right)=\frac{P_{0}}{\text{τ}\left(T\right)}\text{exp}\left[\frac{-1}{\kappa }\overset{T}{\underset{T_{i}}{\int }}\frac{\text{d}T}{\tau \left(T\right)}\right]$$

where *P*_0_ is the initial polarization of the sample, *τ*(*T*) is the relaxation time of the process and *κ* is the heating rate. The relaxation time can be modeled through several empirical models. We use the Arrhenius equation

(4) $$\text{τ}\left(T\right)={\text{τ}}_{0}\text{exp}\left(\frac{E}{RT}\right)$$

and the Narayanaswamy-Moynihan equations

(5) $$\text{τ}\left(T,T_{\text{f}}\right)={\text{τ}}_{0}\text{exp}\left(\frac{xE}{RT}+\frac{\left(1-x\right)E}{RT_{\text{f}}}\right)$$

(6) $$\frac{\text{d}T_{\text{f}}}{\text{d}t}=\frac{T_{\text{f}}-T}{\text{τ}\left(T,T_{\text{f}}\right)}$$

where *E* is the activation energy, τ_0_ the preexponential factor and *T* and *x* (which is related to cooperativity) are empirical parameters.

**Figure 8:**
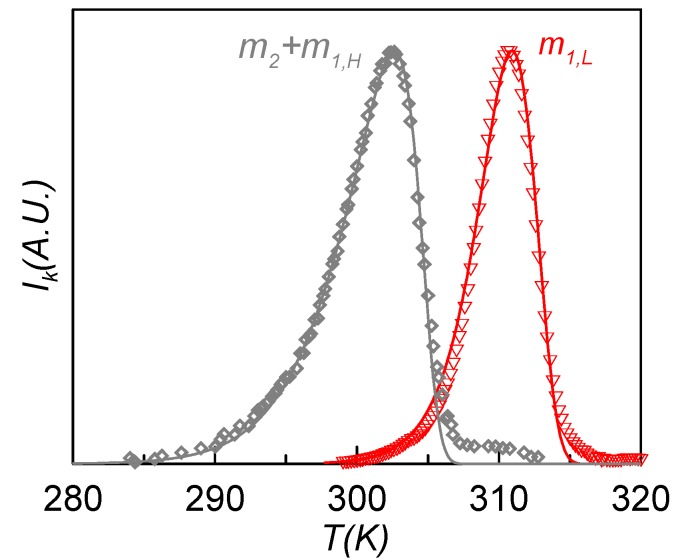
Experimental (symbols) and calculated (lines) TSDC spectra of the m_1L_ (red) and m_2_ + m_1H_ (grey) relaxation modes obtained in the RMA analysis of the sample poled at *T*_p1_ = 308 K (12 MV·m^−1^) and *T*_p1_ = 301 K (1.6 MV·m^−1^).

Figure 8 shows the experimental (symbols) and calculated (lines) TSDC spectra of the m_1L_ (red) and m_2_ + m_1H_ (grey) relaxation modes obtained in the RMA analysis of the sample poled at *T*_p1_ = 308 K (12 MV·m^−1^) and *T*_p1_ = 301 K (1.6 MV·m^−1^), respectively. These curves correspond to the contributions of maximum depolarization response, and so they are the more representative of each relaxation mode. Each depolarization curve of the RMA analysis was fitted to Equation (3) using the Arrhenius and the Narayanaswamy-Moynihan models for the relaxation time dependence. The obtained results show that m_1L_ is best fitted with the Arrhenius equation, while for m_2_ + m_1H_ the best results are obtained with the Narayanaswamy-Moynihan model. This behavior indicates that the m_2_ + m_1H_ relaxation is more cooperative than the m_1L_ one, which agrees with the results obtained from dielectric measurements.

### 2.4. Specific Heat Evidence for Two Glass Transitions

Both dielectric spectroscopy and the RMA from TSDC measurements point to the existence of two glass transitions separated by a temperature gap of about 7 K. When analyzing the specific heat of Figure 1, where the glass transition of one of the confined samples is shown after getting the N glassy state at different cooling rates, no apparent existence of two glass transitions is observed.

**Figure 9:**
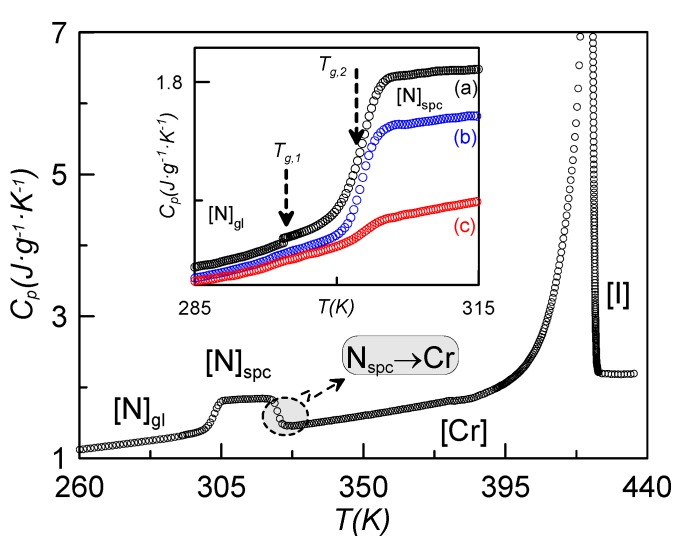
Specific heat *vs.* temperature for the sample of ρ_s_ = 0.23 g·cm^−3^, heating up from 260 K after a fast cooling (20 K·min^−1^). As it can be observed, the data for this sample are qualitatively similar to those shown in Figure 2 for ρ_s_ = 0.16 g·cm^−3^. The inset shows the jump in specific heat coming from several cooling rates: (a) 20 K·min^−1^, (b) 15 K·min^−1^, (c) 10 K·min^−1^.

Figure 9 shows the specific heat on heating the sample ρ_s_ = 0.23 g·cm^−3^ from 260 K after a fast cooling (20 K·min^−1^). As it can be observed, the data for this sample are qualitatively similar to those shown in Figure 2 for ρ_s_ = 0.16 g·cm^−3^. Let us consider the inset of Figure 9, where the jump in the specific heat is shown for the sample coming from several cooling rates. In the case denoted by (a), corresponding to a cooling rate of 20 K·min^−1^, two consecutive glass transitions can be unambiguously observed about 7 K apart. In the other cases, (b) and (c), these two specific heat jumps cannot be clearly observed.

The first sight of Figure 2 apparently leads to the same conclusion. In our opinion, the existence of two glass transition temperatures is independent of the concentration of γ-alumina nanoparticles. First of all, the TSDC spectra for bulk CBO11O.Py is qualitatively similar to that of the confined sample, as can be seen from Figure 7. From specific heat measurements, the cooling rate to achieve the glassy state is a crucial parameter. For high enough concentrations of γ-alumina nanoparticles (ρ_s_ ≥ 0.23 g·cm^−3^), the total vitrification of the N phase is believed to be obtained for cooling rates of 20 K·min^−1^. For lower concentrations of nanoparticles, including the bulk, the highest cooling rate does not seem to be sufficient to avoid the coexistence of a small part of crystal with the nematic glassy state, making the observation of the two glass transition temperatures more difficult.

The different values in the specific heat capacity jumps can be explained as follows: As the pyrene group is considerably bulkier than the cyanobiphenyl one, the m_1L_ mode requires more energy than both m_2_ and m_1H._ So, the jump in the specific heat is more pronounced in the glass transition at *T*_g2_ (=*T*_gPy_).

There are some other systems in which two glass transitions have been found in the same phase [35,36,37], but this is the first time, to the best of our knowledge, that two close glass transitions have been unambiguously identified in the same supercooled mesophase of any liquid crystal.

## 3. Experimental Section

The pure CBO11O.Py compound was synthesized and purified according to the work by Attard *et al*. [24]. The alumina nanoparticles, in the γ-phase, are commercially obtained from Tecnan and their purity were claimed to be 99.995%. The density of the nanoparticles is about 3.65 g·cm^−3^ and they have a surface area of about 110 m^2^·g^−1^. The particles are spherical and have an average diameter length of about 15 nm. The dispersion of the nanoparticles in the pure compound is made mechanically, in an ultrasound bath at temperatures slightly above the N-I transition temperature for the pure compound. This method was proven to be satisfactory for the calamitic monomer 4O.8 [26], and the goodness of calorimetric data, which will be presented below, indicates that this is also the case in the present work.

Static specific heat data at constant pressure were obtained through the Modulated Differential Scanning Calorimetry (MDSC) technique via a commercial TA Instruments Q2000 calorimeter, for which extensive details can be found elsewhere [8,38]. Similar to an AC calorimeter, the MDSC technique, in addition to specific heat data, simultaneously provides phase shift data (Φ) that allow determining the coexistence region in weakly first-order transitions. The experimental conditions were adjusted in such a way that the phase delay (Φ) between the modulated heat flow (the response to the perturbation) and the induced temperature oscillations (perturbation) is nearly zero out of the phase transition and the imaginary part of the complex specific heat data vanishes. Similarly, by means of a special calibration procedure using very precise latent heat data measured from other homologous compounds through adiabatic calorimetry, the MDSC technique is also suitable for quantitative measurements of the latent heat of first order transitions, even when the latent heat is very small. Measurements were performed on cooling from the I phase down to the mesophase and next on heating; the temperature rates on cooling range from 1 to 20 K·min^−1^ and on heating were of 1 K·min^−1^, with a modulation temperature amplitude (temperature oscillations) of ±0.5 K and a period of 60 s.

Measurements of the complex dielectric permittivity were performed with two different pieces of equipment: HP 4291A impedance analyzer for frequencies from 10^6^ Hz to 10^9^ Hz and alpha impedance analyzer from Novocontrol for frequencies from 10^−3^ Hz to 10^6^ Hz. The cell consists of two gold-plated brass electrodes (diameter 5 mm) separated by silica spacers, making a plane capacitor of about 50 μm thick. The sample is held in a cryostat, and the temperature is controlled via a System Quatro from Novocontrol. Additional details of the experimental technique can be found elsewhere [8,38]. Dielectric measurements were performed on heating and on cooling with stabilization at different temperature steps and a temperature control on the order of 20 mK.

The material was polarized with a PLH250-P DC Power Supply (Aim & Thurlby Thandar Instruments, Cambridgeshire, UK) for performing the Thermal Stimulated Depolarization Currents (TSDC) measurements. The depolarization current was registered with a Keithley 6514 System Electrometer. Linkam cells of 5 μm thickness and electrode area of about 1 cm^2^ were used. The cell is placed in a Linkam THMSG-600 hot stage and temperature is controlled via a Linkam TMS-94 temperature controller. At the same time as the TSDC measurements were performed, optical textures were recorded with a Kyowa polarizing microscope.

## 4. Conclusions

The initial and prime interest of the present study was to make a homogeneous hybrid liquid crystal material composed by γ-alumina nanoparticles dispersed in CBO11O.Py liquid crystal dimer. The main functionality of such a material was to suppress crystallization when the sample is cooled down slowly from the isotropic phase with the purpose of studying the dynamics close to the glass transition. It has been proven that for concentrations, ρ_s_, higher than or equal to 0.16 g·cm^−3^, the sample vitrifies at slowly cooling rates of 5 K·min^−1^, but higher concentrations of γ-alumina do not improve the functionality.

The main milestone of the present work was the identification of two glass transition temperatures close to each other by means of several complementary experimental techniques: broadband dielectric spectroscopy, thermal stimulated depolarization current and calorimetry. The former two allow us to identify the molecular motions involved in both glass transitions. It is suggested that the highest temperature glass transition corresponds to the flip-flop motions of the bulky pyrene group, whereas the lowest one is due to both flip-flop and precessional motions of the cyanobiphenyl unit. On the other hand, the authors infer that the existence of two glass transition temperatures is also valid for bulk CBO11O.Py liquid crystal dimer. This conclusion is clearly supported by means of TSDC technique.
